# Study of Foeniculum vulgare Effect on Folliculogenesis in Female Mice

**Published:** 2011-12-22

**Authors:** Mozafar Khazaei, Azadeh Montaseri, Mohammad Rasool Khazaei, Masumeh Khanahmadi

**Affiliations:** 1 Fertility and Infertility Research Center, Kermanshah University of Medical Sciences, Kermanshah, Iran; 2 Department of Chemistry, Kermanshah Branch of ACECR, Kermanshah, Iran

**Keywords:** Foeniculum vulgare, Folliculogenesis, Ovary, Mice

## Abstract

**Background:**

Foeniculum vulgare (FVE) is used in traditional medicine for its antiseptic, palliative and anti-inflammatory effects. Traditionally, FVE is utilized for treating female infertility. The present study aims to investigate the effects of FVE extract on folliculogenesis in female albino mice.

**Materials and Methods:**

In this experimental study, a total of 20 female albino mice were divided into four groups. Groups 1 and 2 (experimental) received FVE alcoholic extract at doses of 100 and 200 mg/kg body weight (BW)/day for five days. Group 3 (negative control) received ethanol and group 4 (positive control) was administered normal saline, in the same doses as the experimental groups. Animals in all groups were sacrificed on the sixth day of the study; their ovaries were dissected out and prepared for histological examinations. Hematoxylin and eosin (H&E) stained microscopic slides were evaluated and the numbers of ovarian follicles were compared between groups. Data were analyzed by one way ANOVA.

**Results:**

The total follicle numbers were 26.5 ± 5.24 for group 1 (100 mg/kg FVE), 27.2 ± 4.1 for group 2 (200 mg/kg FVE), 10.1 ± 2.53 for group 3 (ethanol control) and 17.2 ± 3.9 for the saline control group (group 4). The numbers of graffian, antral and multilaminar follicles increased significantly in both experimental groups when compared with the control groups (p<0.05), however there were no significant differences in follicle numbers among the experimental groups. The number of unilaminar primary follicles did not significantly change between all groups. GCMS analysis of FVE extract identified the presence of diosgenin, an estrogenic compound.

**Conclusion:**

FVE induced folliculogenesis in female mice ovary and increased the number of growing ovarian follicles. The estrogenic component of FVE, diosgenin, may exert this effect.

## Introduction

Infertility is one of the main problems seen in young couples. Due to cost and social problems, some couples seek traditional methods to treat their infertility before meeting with specialists. Since folk medicine plays an important role in individual and community primary health care, the use of herbal products are increasing in many countries (1). At present, great efforts are underway to studies medicinal plants antioxidant and antimicrobial extract both in industry and for use in scientific research (2). It seems medicinal plant properties are related to many phytochemical components, including carotenoids, flavanoids, curcumins and terpenoids (3).

Foeniculum vulgare Mill (FVE; Fam. Umbellifarae), commonly known as fennel, is a small genus of annual, biennial or perennial herbs located in central Europe and the Mediterranean region. It is widely used as a culinary spice and grown in different tropical regions of the world for its aromatic fruits (4). The FVE fruit has a long history of use as both a food and medicine. Traditionally, it is said to act as a carminative (assists with flatulence control) and increase breast milk production. It has been reported to enhance libido, promote menstrual flow, soothe indigestion and cough (5).

FVE has a clear protective effect against ethanolinduced gastric lesions, which is related to decreases in lipid peroxidation and antioxidant activity (6). Additionally, FVE can be used for pediatric colic and some respiratory disorders due to its anti-spasmodic effects (7, 8). It is widely acknowledged that all parts of FVE, including the seed have considerable medicinal value (9). For centuries, FVE fruits have been used as traditional herbal medicines in many Asian countries (10). It is also used as a spice in tropical Asia (11). FVE is used in Indonesia to cure albuminuria, abdominalgia, insomnia, and menstrual disorder, and the seed’s oil is utilized for the treatment of carminative, colds, and asthma (12).

FVE is an indigenous herb in Iran, and the plant extract has been used as an antiseptic, palliative and anti-inflammatory. In a study, FVE essence and mefenamic acid were compared for the treatment of primary dysmenorrheal and it was reported that FVE could be used as a safe and effective herbal drug for primary dysmenorrhea (13).

FVE fruit also possesses emnenagague, galactagogue, carminative, diuretic, and lactation stimulant properties (14). It contains 1% - 3% of a volatile oil, which is composed of 50% - 85% of anethole and about 20% of d-fenchone (15, 16). Other compounds present in the fruit are d-apinene, d-a-phellandrene, dipentene, methyl chavicol , fenelon, anisaldehyde and anisic acid (17).

FVE has been known to be able to regulate menstruation, alleviate the symptoms of female climacteric syndrome, and increase libido (18). One study has shown that FVE seed increases the weight of the genital organs. Oral administration of FVE increases total protein concentration in seminal vesicles and prostate glands in male rats, and increases the weight of mammary glands, oviducts, endometrium, and myometrium in female rats (19). FVE essential oil affects uterine contractions and significantly reduces the intensity of oxytocin and Prostaglandin E2-induced contractions (14). In Iranian folk medicine, it has been claimed that FVE improves sexual function and infertility in women, however there is no documented study to clarify this effect.

This study researches the quantitative aspects of folliculogenesis in female albino mice after administration of FVE fruit extract.

## Materials and Methods

### Plant material and extraction

FVE fruits were collected from the bursa and authenticated by a botanist (School of Pharmacy, Kermanshah University of Medical Sciences). The extract was prepared according to Word of Health Organization (WHO) protocol for preparation of an alcoholic extract (20). Briefly, 100 g of fruit was shed-dried, powdered and added to 1000 ml of 70% ethanol (v/v) and left to macerate at room temperature for 20 hours. The basin was slowly rotated during this time. After filtration, ethanol was evaporated at low pressure at 30°C.

### Acute toxic dose

The intraperitoneal acute toxicity (LD50) of the extract was evaluated in Swiss albino mice as previously described (21). Briefly, five different doses of the extract were administered to five groups of mice (five mice/group). After 24 hours, there were no deaths in the animals that received the plant extract at doses of 1, 10, 100 and 500 mg/kg, and one death reported at a dose of 1000 mg/kg.

### Animals

The Ethical Committee of Kermanshah University of Medical Sciences approved all procedures used in this study.

A total of 20 virgin, female albino mice with the weight range of 25-30 g were used. The animals were fed standard laboratory chow and water during the experiment. We used the Whitten effect for co-cycling animals (22) and controlled vaginal changes for determining estrous cycle (23).

Proestrus mice were divided into four groups (n=5) (24). Animals in experimental groups (1 and 2) were administered FVE at 100 (group 1) and 200 (group 2) mg/kg/day doses (6). Group 3 (negative control) received ethanol as the FVE extract solvent. Group 4 (positive control) received normal saline and was considered the normal group. Saline and ethanol were administered in the same volumes as groups 1 and 2. All agents were administered interaperitoneally for five days (23).

Animals in all groups were sacrificed on the sixth day of the study. The ovaries were dissected out, cleaned of fat and fixed in 4% neutral buffered formaldehyde. They were dehydrated in graded alcohols, cleared in xylene, embedded in paraffin and then serially sectioned at 6µm and stained with hematoxylin and eosin (H&E).We improved Gupta et al. method to study the histology of the ovary (25) All sections from each ovary were stained. Five complete and the largest (mid-ovary) sections were selected. We counted the number of follicles per section and used the mean of five sections for each ovary, and compared them between groups. Follicles were classified into four stages according to the number of granulosa cell (GC) layers around the oocyte. Stage I were unilaminar primary follicles with one GC layer; Stage II were multilaminar primary follicles with more than one GC layer; Stage III consisted of antral follicles with a single medium size or two small cavities; and Stage IV had graffian follicles with a large, well-formed antral cavity.

### Extract analysis

After determining the estrogenic effect of FVE extract, we examined the extract for diosgenin, a plant steroid with estrogenic effect that is the precursor for steroid hormones. The extract was prepared for GC-MS analysis by standard methods (3, 26). GC-MS analysis was carried out using a Hewlett-Packard 6890 with an HP-5 capillary column. The oven temperature was programmed as follows: 250°C for 15 minutes to 270°C at a 1°C/minute increment rate for 1 minute, the temperature increased to 300°C at a 20°C/minute increment rate for 10 minutes; carrier gas was He (1 ml/minute).

### Statistical analysis

Statistical analyses of data were performed using a one-way analysis of variance (ANOVA) and Tukey’s post hoc test. A value of p<0.05 was considered statistically significant.

## Results

**Fig 1:**
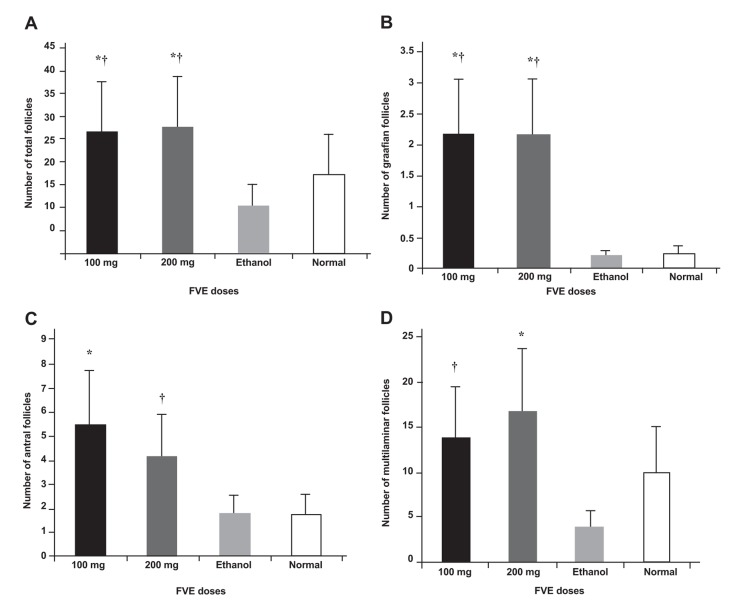
A. Comparison of total number of follicles (graffian, antral and primary) in experimental and control groups. FVE groups showed significant difference with both controls, B. Significant difference in the number of graffian follicles between experimental and control groups, C. Comparison of number of antral follicles in different groups. 100 mg/kg of FVE was the highest number, D. Comparison of number of multilaminar follicles in different groups. 200 mg/kg FVE has the highest number. *p<0.005 vs. ethanol, †p<0.05 vs. normal.

The total number of follicles were 26.5 ± 5.24 in group 1(100 mg/kg FVE), 27.2 ± 4.1 in group 2 (200 mg/kg FVE), 10.1 ± 2.53 in group 3 (ethanol) and 17.2 ± 3.9 in group 4(saline). There were significant increases in the experimental groups compared to the control groups (p<0.05, Fig 1A). There were no significant differences between the FVE concentrations. Similarly a significant increase was observed in the number of graffian, antral and multilaminar follicles (p<0.05) in the FVE groups, but there was no significant difference between the 100 and 200 mg/kg doses of FVE (Fig 1B, C, D).

**Fig 2:**
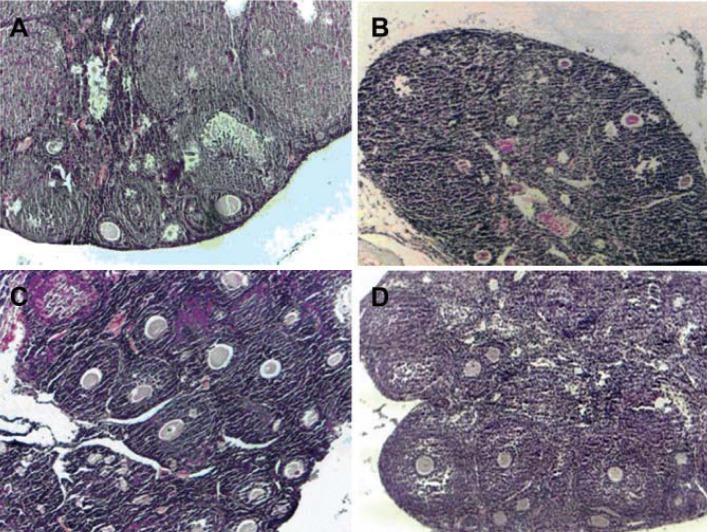
A. Normal histological structure of mouse ovary with different follicles in the cortex. B. Mouse ovary in negative control (ethanol) group with degenerated follicles. C. Numerous growing (multilaminar) follicles in the ovarian cortex of mice in the 100 mg FVE group. D. Mice ovary with numerous growing (multilaminar and antral) follicles in the ovarian cortex in the 200 mg/kg FVE group (×100).

There was no significant difference in the number of unilaminar primary follicles between all experimental and control groups. Histological sections of control and experimental groups showed increased numbers of growing follicles in the FVE groups (Fig 2A-D). GC-MS analysis showed existence of diosgenin, an estrogenic compound in the FVE extract.

## Discussion

To our knowledge, there is no scientific report on the effect of FVE on the ovary. In the present study, the alcoholic extract of FVE fruit has induced a significant increase in the numbers of graffian, antral and multilaminar primary follicles and improved folliculogenesis in mice ovaries. This may explain traditional fennel consumption to improve female infertility. Follicular growth is regulated by endocrine hormones [follicle stimulating hormone (FSH), luteinizing hormone (LH) and prolactin] and local (paracrine and autocrine) factors (27). The significant increase in the number of follicles in the presence of FVE may have been due to the estrogenic effects of this plant.

FVE constituent's dianethole and photoane hole resemble stilbene and diethylstilbestrol, and anethole is structurally similar to cathecolamines, which may influence secretion of prolactin (28). Steroids and prolactin are involved in ovarian follicullogenesis. In this study it has been determined that one of the active constitutes of FVE extract is diosgenin a steroid sapogenin which is the starting material for the synthesis of a number of hormonal products such as DHEA. The estrogenic effects of diosgenin have also been demonstrated (29). In addition, diosgenin has been used to treat osteoporosis in the ovariectomized adult rat model (30).

As described in many studies, FVE can affect some reproductive factors. FVE appears to induce estrus in rats (18). Malini et al. have shown that FVE seed extract had estrogenic activity on the female and male genital organs and increased the weight of genital organs. In female rats, oral administration of FVE for ten days increased the weight of the mammary glands, oviduct, endometrium, myometrium, cervix and vagina (19). The researchers did not study the histological changes of the ovaries and genital organs. Here, we have shown the effect of FVE on the histology of mouse ovaries over a shorter period of time (five days), which emphasized the potent folliculogenesis effect of FVE. FVE also showed radical scavenging and antioxidant properties (31) which can affect general cell growth.

Both FVE doses showed nearly the same folliculogenesis effect, but 100 mg/kg had more numbers of antral follicles (Fig 1C) and 200 mg/kg FVE had more numbers of multilaminar follicles (Fig 1D). We did not utilize a stereological study to calculate all follicle numbers in each ovary. Our method was an improvement of a previous technique reported by other researchers (25, 32). The method consists of a reliable, repeatable technique for studying the effect of herbal extracts on animal ovaries.

## Conclusion

The present study elucidated the fact that FVE has a folliculogenesis effect in female mice consistent with its use in folk medicine as a fertility enhancing agent. Further studies are suggested for understanding the exact mechanism(s) underlying these actions and probable changes in hormonal levels.
